# Screening for lung cancer with Low-Dose Computed Tomography: a systematic review of the evidence

**DOI:** 10.1186/1617-9625-12-S1-A8

**Published:** 2014-06-06

**Authors:** Sotiria-Maria E Iliopoulou, Antonis A Kousoulis

**Affiliations:** 1 Medical School, National and Kapodistrian University of Athens, Athens, 115 27, Greece; 2 Society of Junior Doctors, Athens, 15123, Greece; 3 Department of Public Health, Imperial College London, London, SW7 2AZ, UK

## Background

Tobacco use is the principal risk factor for lung cancer. Lung cancer is the most common cause of cancer death worldwide. When identified clinically, most patients have advanced disease with poor prognosis: the mortality rate at stage IV is over 95.0%, whereas the 5-year survival rate is over 73.0% at stage I. Thus, there is a growing interest in the early detection of lung cancer with Low-Dose Computed Tomography (LDCT) scans. The objective of the study is to conduct a systematic review of the evidence in Randomized Clinical Trials (RCTs) assessing the effect of screening with LDCT on lung cancer mortality and assess benefit and harm.

## Materials and methods

PubMed was our data source (search period: November 2002 to September 2013). Of 796 English citations reviewed, we have included 10 RCTs regarding LDCT screening of high risk individuals for lung cancer.

## Results

The National Lung Cancer Screening Trial recruited 53,454 asymptomatic smokers and ex-smokers between the ages of 55 and 74, with smoking histories of at least 30 pack-years (most guidelines’ target population).It found a 20% reduction in lung cancer mortality (95% C.I.: 6.8 - 26.0, p = 0.004) and a 6.7% reduction in all-cause mortality (95% C.I.: 1.2 - 13.6, p= 0.02) in the 3 annual LDCDs arm compared to the three annual CXRs arm. The smaller European DLCST, MILD, Italung and DANTE trials, with one to five annual LDCTs, fail to reach a statistically significant difference in lung-cancer mortality (DANTE: RR: 0.97;95% CI, 0.71-1.32; p=.84; DLCST:RR, 1.15; 95% CI, 0.83-1.61;p=.43); the same was the case with the MILD trial with one or two annual LDCTs. The small Depiscan trial and the trial by Garg et al. (LDCT vs CXR and no screening respectively) with short follow-up periods reported higher detection rates of non-calcified nodules in the LDCT arm. The biggest so far European clinical trial N.E.L.S.O.N. with three rounds of LDCT screening has the purpose to determine whether CT screening will reduce lung cancer mortality by at least 25%. This, as well as the UKLS trial and the German LUCI trial, is not yet completed.

## Conclusions

There is a big heterogeneity in the findings and the frequency of false positive results (10%-96.4%), the detection rates, the number of LDCTs performed (one to fine annually), the sensitivity of screening (up to 94%), follow-up period (33 months to 10 years), further assessment of nodules, cost-effectivess, types of biases and the grade of compliance among participants. Individuals at high risk of developing lung cancer who meet the criteria for CT should consult their physicians in order to make a conscious decision about following the current guidelines. However, results from new studies will provide further insight.

